# Humanized Transgenic Mice Are Resistant to Chronic Wasting Disease Prions From Norwegian Reindeer and Moose^[Author-notes jiab033-FM1]^

**DOI:** 10.1093/infdis/jiab033

**Published:** 2021-01-27

**Authors:** Jonathan D F Wadsworth, Susan Joiner, Jacqueline M Linehan, Kezia Jack, Huda Al-Doujaily, Helena Costa, Thea Ingold, Maged Taema, Fuquan Zhang, Malin K Sandberg, Sebastian Brandner, Linh Tran, Turid Vikøren, Jørn Våge, Knut Madslien, Bjørnar Ytrehus, Sylvie L Benestad, Emmanuel A Asante, John Collinge

**Affiliations:** Medical Research Council Prion Unit at University College London, University College London Institute of Prion Diseases, London, United Kingdom; Medical Research Council Prion Unit at University College London, University College London Institute of Prion Diseases, London, United Kingdom; Medical Research Council Prion Unit at University College London, University College London Institute of Prion Diseases, London, United Kingdom; Medical Research Council Prion Unit at University College London, University College London Institute of Prion Diseases, London, United Kingdom; Medical Research Council Prion Unit at University College London, University College London Institute of Prion Diseases, London, United Kingdom; Medical Research Council Prion Unit at University College London, University College London Institute of Prion Diseases, London, United Kingdom; Medical Research Council Prion Unit at University College London, University College London Institute of Prion Diseases, London, United Kingdom; Medical Research Council Prion Unit at University College London, University College London Institute of Prion Diseases, London, United Kingdom; Medical Research Council Prion Unit at University College London, University College London Institute of Prion Diseases, London, United Kingdom; Medical Research Council Prion Unit at University College London, University College London Institute of Prion Diseases, London, United Kingdom; Medical Research Council Prion Unit at University College London, University College London Institute of Prion Diseases, London, United Kingdom; Department of Neurodegenerative Disease, University College London Queen Square Institute of Neurology and Division of Neuropathology, National Hospital for Neurology and Neurosurgery, University College London National Health Service Foundation Trust, London, United Kingdom; Norwegian Veterinary Institute, Oslo, Norway; Norwegian Veterinary Institute, Oslo, Norway; Norwegian Veterinary Institute, Oslo, Norway; Norwegian Veterinary Institute, Oslo, Norway; Norwegian Institute for Nature Research, Trondheim, Norway; Norwegian Veterinary Institute, Oslo, Norway; Medical Research Council Prion Unit at University College London, University College London Institute of Prion Diseases, London, United Kingdom; Medical Research Council Prion Unit at University College London, University College London Institute of Prion Diseases, London, United Kingdom

**Keywords:** chronic wasting disease (CWD), moose, prion, prion disease, prion protein, reindeer, transgenic mice, transmissible spongiform encephalopathy (TSE)

## Abstract

Chronic wasting disease (CWD) is the transmissible spongiform encephalopathy or prion disease affecting cervids. In 2016, the first cases of CWD were reported in Europe in Norwegian wild reindeer and moose. The origin and zoonotic potential of these new prion isolates remain unknown. In this study to investigate zoonotic potential we inoculated brain tissue from CWD-infected Norwegian reindeer and moose into transgenic mice overexpressing human prion protein. After prolonged postinoculation survival periods no evidence for prion transmission was seen, suggesting that the zoonotic potential of these isolates is low.

Chronic wasting disease (CWD) is the transmissible spongiform encephalopathy or prion disease affecting the Cervidae family [1]. CWD is a contagious disease in free-ranging and captive cervid populations and considerable human exposure may be occurring in North America through consumption of hunted deer. Current evidence from experimental and epidemiological research suggests that the zoonotic potential of North American CWD prions is low [1, 2]; however, considerable uncertainties remain. Foremost among these is that the number of prion strains propagating CWD is ill-defined and this situation is compounded by the knowledge that novel prion strains with altered host ranges can arise as a result of prion protein (PrP) polymorphisms in both inter- and intraspecies transmissions [1–4]. Consequently, the potential public health risks of emergent CWD prions in new hosts must be evaluated directly.

Until recently, the occurrence of CWD appeared to be geographically restricted to North America and South Korea; however, from 2016 onwards CWD has been reported in Norwegian wild reindeer *Rangifer tarandus* [5], moose *Alces alces* [6], and red deer *Cervus elaphus* [7], with further cases in moose identified in Finland and Sweden [2]. The origin, strain properties, and zoonotic potential of these first European CWD prion isolates remain unknown; however, the neuropathological phenotypes and the molecular strain types of disease-associated, protease-resistant PrP (PrP^Sc^) seen in affected species suggest that CWD in Norwegian reindeer has similarities to North American CWD but may not be caused by an identical strain [5, 8], whereas CWD in Norwegian moose and red deer are novel [2, 6–8]. In this study, to inform on the zoonotic potential of these isolates, we performed transmission studies of brain samples from Norwegian reindeer and moose with CWD to transgenic mice that overexpress 1 or the other of the 2 common polymorphic forms of human PrP, with either methionine (M) or valine (V) at residue 129, on a congenic mouse PrP null background. These mice are highly appropriate models to aid in the risk assessment of newly emerging animal prions as they are susceptible to classical bovine spongiform encephalopathy (BSE) prions from cattle as well as a wide range of sporadic and acquired human prion isolates [9–11].

## METHODS

### Research Governance

All work with cervid samples at University College London (UCL) was conducted in microbiological containment level 3 facilities with strict adherence to safety protocols. Work with mice was performed under approval and license granted by the UK Home Office (Animals (Scientific Procedures) Act 1986), project license number 70/9022, which conformed to UCL institutional guidelines and Animal Research: Reporting of In Vivo Experiments (ARRIVE) guidelines (www.nc3rs.org.uk/ARRIVE/). Human tissues used in this study (Supplementary Figures 1 and 2) were obtained at autopsy with informed consent from patients or their relatives in accordance with UK legislation and the Human Tissue Authority license held by UCL Institute of Neurology. Ethical approval for using human tissues was given by the National Hospital for Neurology and Neurosurgery and the UCL Institute of Neurology Joint Research Ethics Committee (references 03/N036, 03/N038, and 03/N133).

### Cervid Brain Samples

Frozen brain samples from CWD-infected reindeer (16-04-V142) [5] and moose (16-60-P138 and 16-60-P153, moose numbers 1 and 2, respectively, in the study of Pirisinu et al) [6] and from reindeer (16-04-V176) and moose (16-60-P195) tested negative for CWD were imported from the Norwegian Veterinary Institute Oslo to UCL London under license granted by the UK Animal and Plant Health Agency (license No. ITIMP16.0426A).

### Western Blotting

Brain samples were prepared as 10% (w/v) homogenates in Dulbecco’s sterile phosphate buffered saline lacking Ca^2+^ and Mg^2+^ ions (D-PBS) using Duall glass tissue grinders (Anachem) or a Precellys Evolution tissue homogenizer (Bertin Instruments). Proteinase K (PK) digestion (50 or 100 μg/mL final PK concentration in the sample, 1 hour, 37°C) electrophoresis, and immunoblotting was performed as described previously [12]. Samples were analyzed on Novex 16% Tris-Glycine gels (Thermo Fisher Scientific) or 15% Criterion Tris-HCl gels (Bio-Rad Laboratories) and calibrated using the Seeblue Prestained Protein Standard from Invitrogen (Thermo Fisher Scientific). High-sensitivity immunoblot detection of human or cervid PrP was performed using anti-PrP monoclonal antibodies 3F4 (epitope spanning residues 104–113 of human PrP) or ICSM 35 (epitope spanning residues 93–105 of human PrP), respectively. Sodium phosphotungstic acid (NaPTA) precipitation of PrP^Sc^ from 10% (w/v) brain homogenate was performed as described previously [12].

### Transmission Studies

Transgenic mice homozygous for a human PrP 129V transgene array and murine PrP null alleles (*Prnp*^*o/o*^), designated Tg(HuPrP129V^+/+^*Prnp*^*o/o*^)-152c mice (129VV Tg152c mice), or homozygous for a human PrP 129M transgene array and murine PrP null alleles (*Prnp*^*o/o*^), designated Tg(HuPrP129M^+/+^*Prnp*^*o/o*^)-35c mice (129MM Tg35c mice) have been described previously [10, 11]. Both lines of mice have a congenic FVB/N, mouse PrP null, background and were derived from 129MM Tg35 and 129VV Tg152 parental lines, which have been used extensively by us in previous human and animal prion transmission studies [9]. 129MM Tg35c and 129VV Tg152c overexpress human PrP in brain at levels of 2- and 6-times that of pooled human brain, respectively. The genotype of each mouse was confirmed by polymerase chain reaction (PCR) of ear punch DNA before inclusion and all mice were uniquely identified by subcutaneous transponders. Disposable cages were used and all cage lids and water bottles were also uniquely identified by transponder and remained with each cage of mice throughout the incubation period. Mice (female, aged 6–8 weeks) were randomly assigned to experimental groups of 20 and anaesthetized with a mixture of halothane and O_2_, and intracerebrally inoculated into the right parietal lobe with 30 µL of 1% (w/v) brain homogenate in D-PBS. Thereafter all mice were examined daily for early indicators of clinical prion disease including piloerection, sustained erect ears, intermittent generalized tremor, unsustained hunched posture, rigid tail, mild loss of coordination, and clasping hind legs when lifted by the tail. Definite diagnosis of clinical prion disease (triggering experimental end point) is reached if mice exhibit any 2 early indicator signs in addition to 1 confirmatory sign, or any 2 confirmatory signs. The confirmatory signs included ataxia, impairment of righting reflex, dragging of hind limbs, sustained hunched posture, or significant abnormal breathing. As clinical diagnosis can be confounded by nonspecific conditions that develop in mice as they age, we limited these confounding effects by electively culling mice after a defined postinoculation period of 700 days. Such elective culling reduces the occurrence of “found dead” mice that die of old age, in which autolytic deterioration of brain tissue often precludes immunohistochemistry (IHC) analyses. At post mortem, brains from inoculated mice were removed, divided sagittally with half frozen and half fixed in formal-saline.

### Neuropathology and Immunohistochemistry for Detection of PrP

Transgenic mouse brains fixed in 10% buffered formal-saline were immersed in 98% formic acid for 1 hour and paraffin wax embedded. Following further washing in 10% buffered formal-saline, tissue samples were processed and paraffin wax embedded. Serial sections of 4 μm nominal thickness were taken. Deparaffinized sections were investigated for abnormal human PrP deposition on a Ventana Discovery XT automated IHC staining machine (Roche Tissue Diagnostics) using protocols developed on a Ventana Benchmark staining machine [12]. Briefly, sections were treated with cell conditioning solution (Discovery CC1; Roche Tissue Diagnostics) at 95°C for 30 minutes followed by treatment with a low concentration of protease (Protease 3; Roche Tissue Diagnostics) for 12 minutes. Anti-PrP monoclonal antibodies 3F4 and ICSM 35 were used in conjunction with biotinylated polyclonal rabbit anti-mouse immunoglobulin secondary antibodies (Dako; Agilent) and Ventana proprietary detection reagents utilizing 3,3′-diaminobenzidine tetrahydrochloride as the chromogen (DAB Map Detection Kit; Roche Tissue Diagnostics). Conventional methods on a Gemini AS Automated Slide Stainer (Thermo Fisher Scientific) were used for hematoxylin and eosin (H&E) staining. Positive controls for the staining technique were used throughout. All slides were digitally scanned on a LEICA SCN400 instrument, and images were captured from the LECIA slidepath software and composed with Adobe Photoshop.

## RESULTS

**Figure 1.**
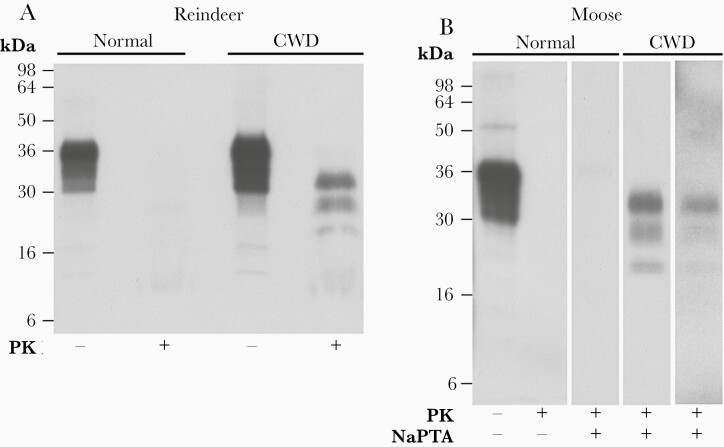
Immunoblot analyses of CWD-negative and CWD-infected Norwegian reindeer and moose brain. *A* and *B*, Immunoblots of brain homogenates analyzed with anti-PrP monoclonal antibody ICSM 35 and high-sensitivity enhanced chemiluminescence. *A*, Brain homogenates, 10% (w/v), from a CWD-negative reindeer (16-04-V176; normal) and CWD-infected reindeer (16-04-V142; CWD) analyzed before (−) or after (+) digestion with PK (50 µg/mL final protease concentration, 1 hour, 37°C). The provenance of the brain sample is designated above each lane. Loading volumes for the immunoblot were 2.5 µL of PK− and 10 µL of PK+ 10% (w/v) brain homogenate. *B*, Brain homogenates, 10% (w/v), from a CWD-negative moose (16-60-P195; normal), and CWD-infected moose (16-60-P153 and 16-60-P138; CWD). The provenance of the brain sample is designated above each lane. Samples were analyzed with (+) or without (−) PK digestion (50 µg/mL final protease concentration, 1 hour, 37°C) and with (+) or without (−) preconcentration by NaPTA precipitation (from 100 µL 10% (w/v) brain homogenate). The volume equivalent of 10% (w/v) brain homogenates loaded for the immunoblots was 5 µL of PK−/NaPTA−, 5 µL of PK+/NaPTA−, and 100 µL of PK+/NaPTA+. The normal cellular isoform of PrP (PrP^C^) present in both normal and CWD-infected brain homogenate is completely degraded by PK, whereas the scrapie or disease-associated isoform of PrP (PrP^Sc^) comprises aggregated assemblies with the C-terminal two-thirds of the protein showing marked resistance to proteolytic degradation, generating 3 bands corresponding to N-terminally truncated fragments of di-, mono-, and nonglycosylated PrP. The concentration of PrP^Sc^ in 10% (w/v) brain homogenate from CWD-infected moose 16-60-P153 was about 5-fold higher than CWD-infected moose 16-60-P138. Exposure times were varied to show comparable signal strength. Abbreviations: CWD, chronic wasting disease; NaPTA, sodium phosphotungstic acid; PK, proteinase K; PrP, prion protein.

Cervid brain samples imported from the Norwegian Veterinary Institute were prepared as 10% (w/v) homogenates in sterile D-PBS and analyzed for detectable PrP^Sc^ by western blotting to access their suitability for transmission studies. While PrP^Sc^ was readily detected by direct analysis of 10 µL 10% (w/v) CWD-infected reindeer brain homogenate (Figure 1) the two 10% (w/v) CWD-infected moose brain homogenates had lower relative levels of PrP^Sc^ (approximately 10- or 50-fold less than reindeer brain homogenate) that required NaPTA precipitation from a larger volume (100 µL) for reliable detection (Figure 1). Despite lower levels of PrP^Sc^, these moose brain homogenates are highly appropriate for transmission studies as peripheral tissues from animals with CWD, that would most likely be consumed by humans, typically contain PrP^Sc^ at concentrations orders of magnitude lower than present in the central nervous system [13].

PrP^Sc^-positive 10% (w/v) CWD-infected reindeer and moose brain homogenates were diluted to 1% (w/v) with sterile D-PBS and 30 µL inoculated intracerebrally into groups of 129MM Tg35c mice and 129VV Tg152c mice. Mice were then examined daily over postinoculation periods up to 700 days and killed if they were exhibiting signs of distress or if a diagnosis of clinical prion disease was established. To determine whether recipient mice had become prion infected, frozen brain samples were prepared as 10% (w/v) brain homogenates in D-PBS and analyzed for detectable PrP^Sc^ by NaPTA precipitation and high-sensitivity western blotting, and fixed brain samples were analyzed for neuropathological changes and abnormal PrP deposition by IHC.

Despite prolonged postinoculation periods extending up to 700 days, Norwegian CWD prion isolates produced no clinical prion disease, or biochemical evidence (Supplementary Figure 1) or histopathologic evidence (Supplementary Figure 2) for subclinical prion infection in any inoculated mouse (Table 1). The lack of detection of disease-related PrP in the brains of inoculated mice by either high-sensitivity immunoblotting or IHC indicates that both the methionine and valine 129 polymorphs of human PrP are highly resistant to pathological conversion by CWD prions from these Norwegian reindeer and moose.

**Table 1.** Survival Times of Human PrP-Expressing Transgenic Mice Inoculated With CWD Prions From Norwegian Reindeer and Moose

Inocula		Transmission			
		129MM Tg35c Mice		129VV Tg152c Mice	
Prion Agent	Source Code	Attack Rate^a^	Survival, d^b^	Attack Rate^a^	Survival, d^b^
Reindeer CWD	16-04-V142	0/19	373, 391, 457, 509, 518, 523, 630–700 (13)	0/17	498, 513, 519, 553, 567, 629–700 (12)
Moose CWD	16-60-P138	0/20	210, 258, 263, 390, 425, 428, 441, 475, 502, 524, 533, 566, 587, 634–700 (7)	0/20	253, 370, 378, 454, 539, 592, 600–700 (14)
Moose CWD	16-60-P153	0/19	409, 423, 553, 601–700 (16)	0/19	464, 482, 526, 539, 539, 589, 596, 609–700 (12)

Abbreviations: CWD, chronic wasting disease; PrP, prion protein.

^a^Groups of 20 mice were inoculated intracerebrally with 30 µL of 1% (w/v) brain homogenate. Attack rate is defined as the total number of both clinically affected and subclinically infected mice as a proportion of the number of inoculated mice. Subclinical prion infection was assessed by immunohistochemical examination of brain for abnormal PrP deposition and by sodium phosphotungstic acid precipitation of 250 µL 10% (w/v) brain homogenate and analysis for protease-resistant PrP by proteinase K digestion and immunoblotting.

^b^The interval between inoculation and culling due to intercurrent illness, senescence, or termination of the experiment at 700 days postinoculation. Death dates of individual mice are shown together with the range for mice surviving beyond 600 days with the number of mice in this range shown in parentheses. Mice culled with postinoculation periods of ≤ 200 days due to intercurrent illness (all confirmed negative for subclinical prion infection) were not included in calculating attack rates.

## Discussion

The zoonotic transmission of BSE prions from cattle has dramatically highlighted the potential risk posed to humans from dietary exposure to animal prions. In this study we found that transgenic mice overexpressing 1 or the other of the 2 common residue 129 polymorphic forms of human PrP, which are susceptible to cattle BSE prions, are highly resistant to infection with recently identified CWD prions from Norwegian reindeer and moose. These data are congruent with previous findings that our human PrP transgenic mice are highly resistant to infection with North American CWD prions from mule deer [14] and with other studies using different lines of humanized transgenic mice that showed no prion transmission after challenge with North American CWD isolates derived from elk, mule deer, or white-tailed deer (reviewed in reference [2]). Although certain North American CWD isolates appear to have the capacity to seed the misfolding of human PrP in vitro (see reference [2] for review), in vivo studies performed with humanized mice and macaques are considered to be the most pertinent models of human susceptibility [2].

The apparent inability of prions from Norwegian reindeer and moose to propagate in intracerebrally inoculated transgenic mice overexpressing human PrP is reassuring and suggests that the potential zoonotic threat from the new European isolates investigated here is low and not overtly dissimilar to any of the North American CWD isolates so far examined in humanized transgenic mouse models [2]. However, determining whether transmission barriers in our mice are absolute will now require secondary passage of brain homogenates from “negative” challenged mice from this experiment in further transgenic mice. In addition, because splenic PrP expression levels in our transgenic mice are low, we did not collect this tissue for analyses. Findings in spleen from ongoing bioassays of both Norwegian and North American CWD isolates in other lines of humanized transgenic mice will be important to see whether lymphoid tissue expressing human PrP is permissive to CWD prion replication [2]. Finally, because polymorphisms of PrP can dictate prion strain selection [1–4], and reindeer PrP in particular is highly polymorphic [15], it will be of critical importance to evaluate the zoonotic potential of further European CWD cases with novel neuropathological features or divergent PrP genotypes as they emerge.

## Supplementary Data

Supplementary materials are available at *The Journal of Infectious Diseases* online. Consisting of data provided by the authors to benefit the reader, the posted materials are not copyedited and are the sole responsibility of the authors, so questions or comments should be addressed to the corresponding author.

jiab033_suppl_Supplementary_Figure_1

jiab033_suppl_Supplementary_Figure_2

## Notes

***Acknowledgments.*** We thank our biological services team for animal care and R. Newton for preparation of figures. We are very grateful to patients and their families for generously consenting to use of human tissues in this study.

***Financial support.*** This work was supported by the Norwegian Ministry of Agriculture (Norwegian Chronic Wasting Disease Surveillance Program grant to Norwegian Veterinary Institute and Norwegian Institute for Nature Research); Norwegian Veterinary Institute (grant number 12081-CWD); and the Medical Research Council, UK (grant numbers MC_U123160655 and MC_UU_00024/5 to University College London).
