# Supplementary material for: Humanized Transgenic Mice Are Resistant to Chronic Wasting Disease Prions From Norwegian Reindeer and Moose
Source: J Infect Dis. 2021 Jan 27;226(5):933–7. doi: 10.1093/infdis/jiab033 (PMC9470110; doi:10.1093/infdis/jiab033)
Supplement: jiab033_suppl_Supplementary_Figure_1 [file jiab033_suppl_supplementary_figure_1.docx]

**Wadsworth et al J Infect Dis**

**Supplementary Figure 1**


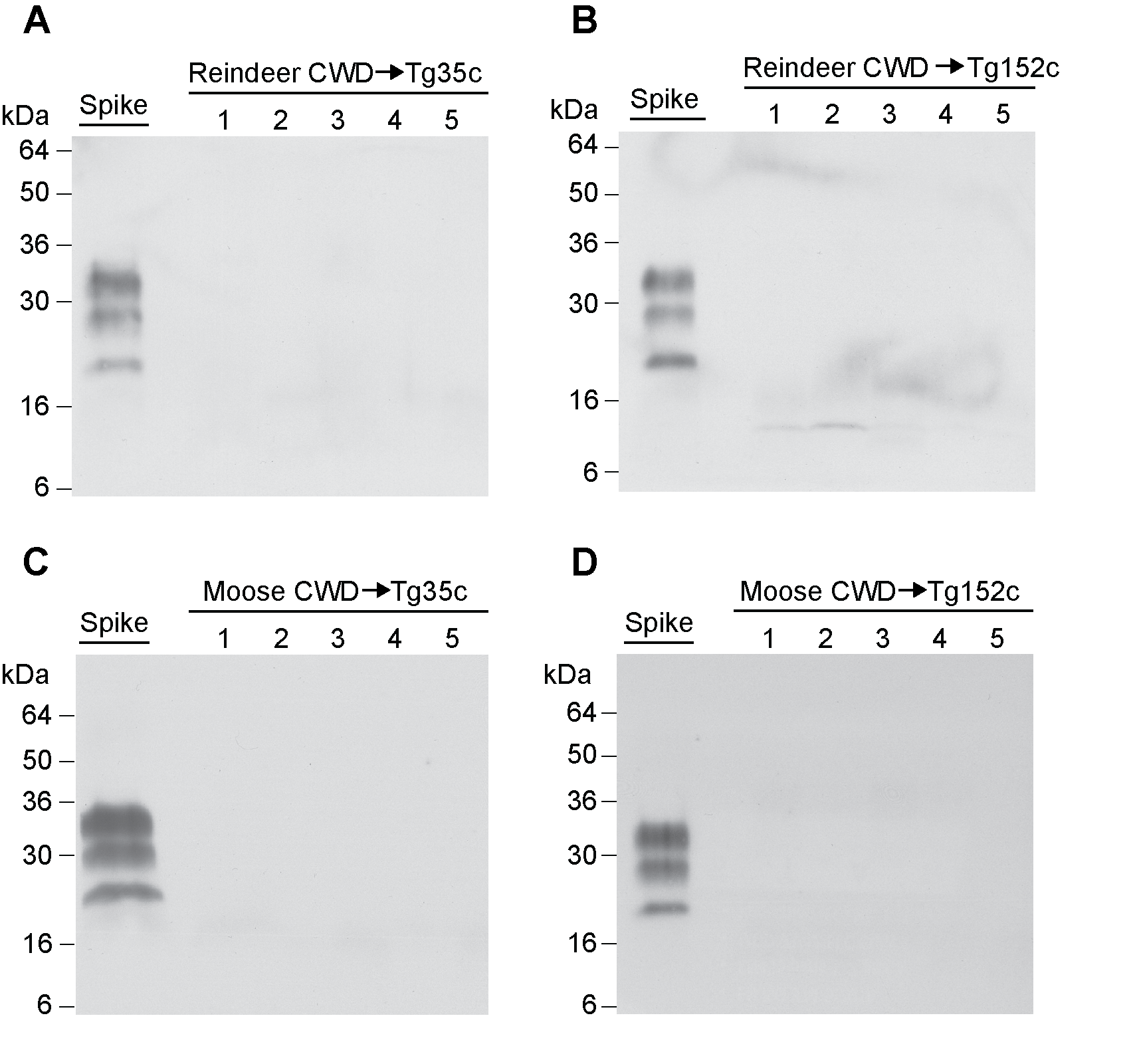


**No detection of PrP^Sc^ in the brain of transgenic mice inoculated with CWD prions from Norwegian reindeer and moose.** Panels A-D show representative immunoblots using anti-PrP monoclonal antibody 3F4 (epitope spanning residues 104-113 of human PrP) and high sensitivity enhanced chemiluminescence. All lanes contain sodium phosphotungstic acid (NaPTA) pellets recovered from 250 µl 10 % (w/v) transgenic mouse brain homogenate treated with proteinase K (PK) (100 µg/ml final protease concentration, 1 h, 37°C). All panels have a positive control (spike) showing efficient recovery of PrP^Sc^ after spiking 0.25 μl 10 % (w/v) vCJD patient brain homogenate into 250 μl of 10% (w/v) normal Tg35c mouse brain homogenate. Each panel shows five brain samples from our transmission series to Tg35c or Tg152c transgenic mice (Table 1). (A, B) Mice challenged with CWD prions from Norwegian reindeer 16-04-V142. (C, D) Mice challenged with CWD prions from Norwegian moose 16-60-P138. The provenance of the brain samples are designated above each lane. PrP^Sc^ in the PK-digested spiked samples comprises three bands corresponding to N-terminally truncated forms of di-, mono-, and non-glycosylated PrP. Weak bands of ~12 kDa seen in lanes 1 and 2 of panel B are non-specific and due to cross-reactivity with the secondary antibody.
