# Supplementary material for: Humanized Transgenic Mice Are Resistant to Chronic Wasting Disease Prions From Norwegian Reindeer and Moose
Source: J Infect Dis. 2021 Jan 27;226(5):933–7. doi: 10.1093/infdis/jiab033 (PMC9470110; doi:10.1093/infdis/jiab033)
Supplement: jiab033_suppl_Supplementary_Figure_2 [file jiab033_suppl_supplementary_figure_2.docx]

**Wadsworth et al J Infect Dis**

**Supplementary Figure 2**


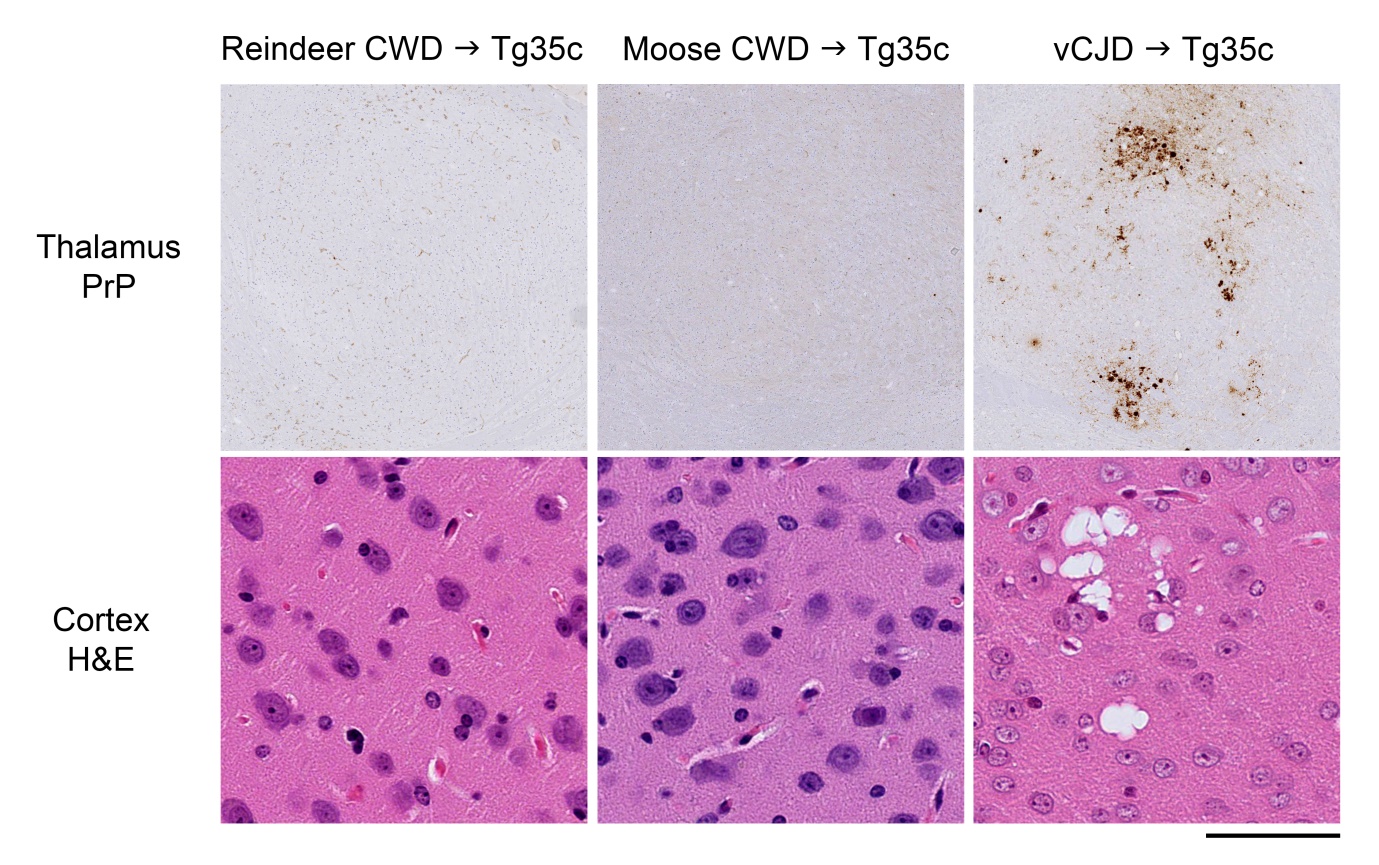


**No detection of abnormal PrP deposition or spongiform neurodegeneration in the brain of transgenic mice inoculated with CWD prions from Norwegian reindeer and moose.** The upper panels show representative PrP immunohistochemistry in the thalamus using anti-PrP monoclonal antibody ICSM 35 (epitope spanning residues 93-105 of human PrP) (PrP) and lower panels show representative haematoxylin- and eosin-staining (H&E) in the cortex to reveal spongiform neurodegeneration. Left panels, unaffected Tg35c mouse (ID 704930) inoculated with CWD prions from Norwegian reindeer 16-04-V142 and culled at 700 days post-inoculation. Centre panels, unaffected Tg35c mouse (ID 708294) inoculated with CWD prions from Norwegian moose 16-60-P153 and culled at 700 days post-inoculation. Right panels, positive control from a subclinically affected Tg35c mouse (ID 330629) inoculated with vCJD prions from human brain and culled at 506 days post-inoculation (transmissions reported in reference 11). Scale bar: upper panels PrP 500 µm; lower panels H&E 50 µm.
